# The Role of the Voltage-Gated Potassium Channel Proteins Kv8.2 and Kv2.1 in Vision and Retinal Disease: Insights from the Study of Mouse Gene Knock-Out Mutations

**DOI:** 10.1523/ENEURO.0032-19.2019

**Published:** 2019-02-25

**Authors:** Nathan S. Hart, Jessica K. Mountford, Valentina Voigt, Paula Fuller-Carter, Melanie Barth, Jeanne M. Nerbonne, David M. Hunt, Livia S. Carvalho

**Affiliations:** 1 School of Biological Sciences, The University of Western Australia, Perth, Western Australia 6009, Australia; 2 Centre for Ophthalmology and Vision Science, Lions Eye Institute, The University of Western Australia, Perth, Western Australia 6009, Australia; 3 Department of Developmental Biology, Washington University School of Medicine, St. Louis, MO 63110

**Keywords:** cone dystrophy, electroretinogram, photoreceptors, potassium channels, retina, retinal degeneration

## Abstract

Mutations in the *KCNV2* gene, which encodes the voltage-gated K^+^ channel protein Kv8.2, cause a distinctive form of cone dystrophy with a supernormal rod response (CDSRR). Kv8.2 channel subunits only form functional channels when combined in a heterotetramer with Kv2.1 subunits encoded by the *KCNB1* gene. The CDSRR disease phenotype indicates that photoreceptor adaptation is disrupted. The electroretinogram (ERG) response of affected individuals shows depressed rod and cone activity, but what distinguishes this disease is the supernormal rod response to a bright flash of light. Here, we have utilized knock-out mutations of both genes in the mouse to study the pathophysiology of CDSRR. The Kv8.2 knock-out (KO) mice show many similarities to the human disorder, including a depressed a-wave and an elevated b-wave response with bright light stimulation. Optical coherence tomography (OCT) imaging and immunohistochemistry indicate that the changes in six-month-old Kv8.2 KO retinae are largely limited to the outer nuclear layer (ONL), while outer segments appear intact. In addition, there is a significant increase in TUNEL**-**positive cells throughout the retina. The Kv2.1 KO and double KO mice also show a severely depressed a-wave, but the elevated b-wave response is absent. Interestingly, in all three KO genotypes, the c-wave is totally absent. The differential response shown here of these KO lines, that either possess homomeric channels or lack channels completely, has provided further insights into the role of K^+^ channels in the generation of the a-, b-, and c-wave components of the ERG.

## Significance Statement

Heterotetrameric Kv voltage-gated K^+^ channels are formed by two subunits, Kv8.2 and Kv2.1, encoded, respectively, by the *KCNV2* gene and *KCNB1* gene. Kv8.2 subunits act as modifiers of channel activity but are not capable of forming functional homomeric channels. Mutations in *KCNV2* result in a blinding disorder that combines photoreceptor dystrophy with an enhanced rod electroretinogram (ERG), a feature that is generally diagnostic for the disorder. In this study, mouse models with knock-out mutations of the *Kcnv2* and *Kcnb1* genes have been used to study of the function of Kv channels in the response to light stimulation as analyzed by ERG recordings to further our understanding of the role of these channels in vision and disease.

## Introduction

We previously demonstrated that mutations in the *KCNV2* gene, which encodes the voltage-gated K^+^ channel protein Kv8.2, are responsible for a distinctive form of cone dystrophy with a supernormal rod response (CDSRR; OMIM 610356; Wu et al., 2006). CDSRR is an inherited autosomal recessive disorder that causes lifelong visual loss. The dystrophy is characterized by reduced visual acuity, photoaversion, night-blindness, and abnormal color vision (Gouras et al., 1983; Sandberg et al., 1990; Kato et al., 1993; Rosenberg and Simonsen, 1993; Hood et al., 1996; Michaelides et al., 2005; Zobor et al., 2012). The electroretinogram (ERG) of affected individuals shows depressed rod and cone activity, but what distinguishes this disease from all other retinal dystrophies is the supernormal rod response to a bright flash of light (Tanimoto et al., 2005); the a-wave response remains reduced and delayed whereas the b-wave becomes supernormal in amplitude. In contrast, cone ERGs are markedly reduced and delayed over the entire range of light intensities. These changes in the ERG are diagnostic for CDSRR (Grigg et al., 2013).

More than 37 different *KCNV2* mutations have now been identified (Gouras et al., 1983; Sandberg et al., 1990; Kato et al., 1993; Rosenberg and Simonsen, 1993; Hood et al., 1996; Michaelides et al., 2005; Wu et al., 2006; Thiagalingam et al., 2007; Ben Salah et al., 2008; Wissinger et al., 2008; Robson et al., 2010; Fujinami et al., 2013), which include missense, nonsense and deletion changes. In all cases, the disorder arises either from the absence of Kv8.2 subunits or, depending on the particular amino acid substitution, from mutant subunits that either fail to be transported to the cell membrane or form non-functional channels (Smith et al., 2012).

Kv8.2 is a member of the Kv2 or *Shab* subfamily of channel subunits that are able to form functional channels only when combined with another channel protein; for Kv8.2, the partner subunit is Kv2.1 encoded by the *KCNB1* gene (Czirják et al., 2007; Smith et al., 2012). A recent study (Gayet-Primo et al., 2018) has shown that both Kv2.1 and Kv8.2 are expressed in the inner segments of photoreceptors. In heterotetramers with Kv2.1 subunits, the role of Kv8.2 subunits is to modulate the activity of the channel. Two disease mechanisms were identified by Smith et al. (2012): pore mutations that result in the formation of non-conducting heteromeric Kv2.1/Kv8.2 channels, and mutations localized in the tetramerization domain of Kv8.2 that prevent the formation of heteromeric channels. With the first mechanism, the heterotetrameric channels would be non-functional, whereas with the second mechanism, only homotetrameric Kv2.1 channels would be formed. Both classes of mutations are recessive and as only minor clinical differences have been observed between patients (Zobor et al., 2012), it would appear that both effectively act as nulls.

The disease phenotype indicates that mutations in *KCNV2* disrupt photoreceptor adaptation, the fundamental physiologic process by which photoreceptor sensitivity is modulated under different levels of illumination. A previous study (Beech and Barnes, 1989) indicated that voltage-gated K^+^ channels in the retina are responsible for a permanent outward K^+^ current, *I_Kx_*. Compared to homomeric Kv2.1 channels, heteromeric Kv2.1/Kv8.2 channels show a reduced inactivation from the open state, less inactivation from intermediate closed states, and a faster recovery from inactivation (Smith et al., 2012). The reduced inactivation from the open state is thought to allow a prolongation of opening in response to sustained depolarizations (Czirják et al., 2007; Mohapatra et al., 2009; Smith et al., 2012).

Since all reported *KCNV2* mutations show a fully recessive mode of inheritance and the clinical features of CDSRR appear to be essentially the same irrespective of the disease-causing mutation, the generation of a gene knock-out of this channel subunit is likely to produce a useful model of this disorder. In this paper, we examine the effects of the loss of Kv8.2 and Kv2.1 subunits on retinal electrophysiology and morphology using knock-out (KO) mouse models of *Kcnv2* and *Kcnb1*.

## Materials and Methods

### Generation of animal lines

The Kv2.1 KO line was generated by the electroporation into mouse ES cells of a targeting construct consisting of a 5′ fragment comprising 2623 bps of *Kcnb1* genomic DNA that terminated at the first base of codon 346, inserted upstream of an IRES-LacZ-Neo cassette, followed by 2457 bps of *Kcnb1* genomic DNA from the first base of codon 380 codon of the *Kcnb1* gene (Jacobson et al., 2007). The targeting cassette removes 102 bases of the *Kcnb1* coding sequence and adds nearly 7 kB of extraneous sequence including a premature stop codon. Western blot analysis of brain tissue confirmed the absence of Kv2.1 protein in homozygous Kv2.1^-/-^ KO mice (Jacobson et al., 2007). This line is on a C57BL/6N background and has been screened for the Rd8 mutation.

**Figure 1.**
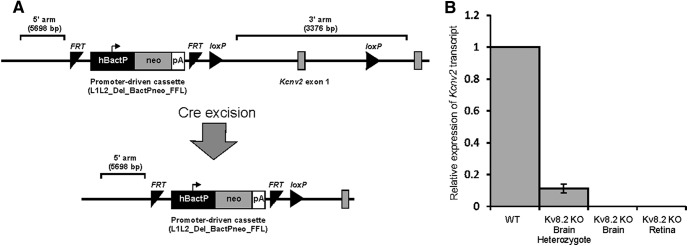
***A***, Targeting and excision of exon 1 of the *Kcnv2* gene. ***B***, qRT-PCR determination of the expression of the *Kcnv2* gene in WT, Kv8.2 KO heterozygous and homozygous mice. Note complete absence of *Kcnv2* expression in Kv8.2 KO homozygous tissues. Error bars indicate SEM. hBactP, human beta actin promoter; neo, neomycin; FRT, flip-recombinase targets.

The Kv8.2^-/-^ KO line was generated through the use of a custom allele targeted to exon 1 of *Kcnv2* in embryonic stem (ES) cells at the Wellcome Trust Sanger Institute (Skarnes et al., 2011). These ES cells were used to produce the *Kcnv2^tm1^* mutant mouse on a standard C57BL/6N background. The *tm1* allele has a custom promoter-driven cassette targeted to the 5’ upstream intron adjacent to exon 1, with LoxP sites flanking this critical exon (Fig. 1). To delete the exon in the *Kcnv2* mutant mice, they were crossed to a ubiquitously expressing CMV-Cre line with the X-linked *Hprt^Tg(CMV-Cre)Brd^* allele on the same C57BL/6N background. Once the confirmation of an excision event was obtained by short range PCR, the CMV-Cre was bred out and animals checked for the *Rd8* mutation. Heterozygous matings were then set up to produce homozygotes; the absence of exon 1 in these mice was confirmed using primers designed to the exon. In wild-type (WT) and heterozygous mice, PCR generated a fragment of the expected size but this fragment was completely absent from homozygotes, indicating that the exon had been deleted in these mice. In addition, a LoxP PCR product generated from heterozygote and homozygote DNA, was reduced in size following the CMV-Cre cross, indicating excision had taken place.

To confirm the absence of any *Kcnv2* transcripts in mice homozygous for the deleted allele, RT-PCR was performed using cDNA generated from RNA extracted from brain tissue at 8 weeks of age. RNA was extracted using TRIzol reagent (Life Technologies, catalog number 15596-026) following the manufacturer’s instructions and qRT-PCR was conducted with TaqMan probe for *Kcnv2* (Thermo Fisher Scientific, Mn00807577_m1) using reagents from Applied Biosystems, following the manufacturer’s instructions. In total, three WT, three homozygous KO, and one heterozygous KO mice were used. The results showed a reduction in the expression of *Kcnv2* in the heterozygote and a complete absence of *Kcnv2* mRNA in the homozygotes (Fig. 1). A second qRT-PCR was then used to confirm the absence of *Kcnv2* transcripts in retinal tissue of Kv8.2 KO mice, using retinal RNA extracted with an RNeasy kit (Qiagen) according to the manufacturer’s instructions. A total of 500 ng of RNA was reverse transcribed using the QuantiTect Reverse Transcription kit (Qiagen), and real-time PCR was performed in triplicate using the TaqMan Fast Advanced Master Mix (Thermo Fisher Scientific) on a Bio-Rad CFX96 Real-Time PCR System. TaqMan Gene Expression Assays (Thermo Fisher Scientific, mouse genes) were used to assess the relative transcript levels of *Kcnv2* (Mm00807577_m1) in WT and Kv8.2 KO mice, normalized to *Gapdh* (Mm99999915_g1). The results confirmed the complete absence of *Kcnv2* transcripts in the retinae of Kv8.2 KO mice.

### Animal housing and ethics

All mice were bred and housed in specific pathogen free conditions at the University of Western Australia (UWA) Biomedical Research Animal Facility. All animal experimentation was performed with the approval of the UWA Animal Ethics Committee, according to the guidelines of the National Health and Medical Research Council of Australia and the Australian Code for the Care and Use of Animals for Scientific Purposes. All mice were group-housed in a climate-controlled facility on a 12/12 h light/dark cycle with food and water *ad libitum*. All experiments were performed on female animals at six months of age unless otherwise stated.

### Optical coherence tomography

A Bioptigen spectral domain ophthalmoscope was used to image the retinae of six-month-old WT and KO mice. Anesthesia was achieved by intrapertioneal administration of a mixture of ketamine (0.167 mg/100 g body weight) and xylazine (0.033 mg/100 g body weight). Pupils of anaesthetized mice were dilated by application of 2.5% phenylephrine hydrochloride and 1% tropicamide drops (Alcon).

### Immunohistochemistry and microscopy

Eyes were collected from WT and KO mice at one, three, and six months of age for analysis of cell death by TUNEL assay (ApopTag Red *In situ* Apoptosis Detection kit, Millipore/Merck). Briefly, eyes were fixed in 4% PFA for 1 h on ice. They were then incubated in 20% sucrose overnight at 4°C. The following day eyes were frozen in optimal cutting temperature compound (Tissue-Tek O.C.T. Compound, Emgrid) and stored at –20°C before sectioning. Retinal sections were collected on superforst slides (Hurst) and cut using a Leica Cryostat CM3050 at 14 µm. TUNEL staining was performed as protocol and TUNEL-positive cells were counted using an Olympus Fluorescent Microscope BX60. Eyes at six months were also collected for gross retinal morphology analysis by hematoxylin and eosin (H & E) staining. These eyes were processed for paraffin sections as follows: eyes were fixed in Davidson media for 24 h at room temperature (RT). They were then incubated for 24 h in 10% formalin and embedded in paraffin. Sections were cut at 20 µM and stained with H & E. Images were taken on an Olympus Fluorescent Microscope BX60.

The number of cone photoreceptors was counted in retinal wholemounts from six-month-old WT and Kv8.2 KO mice labeled with cone arrestin antibody (Millipore catalog #AB15282, RRID:AB_1163387). Dissected retinae were blocked in 1% BSA, 3% Triton X-100, and 5% NGS for 1 h at RT, then incubated for 12–16 h at 4°C with primary antibody (1:1000). The following day, retinae were washed in 1× PBS and incubated for 12–16 h at 4°C in a mix of DAPI solution and Alexa Fluor 546-conjugated secondary antibody (1:500). Retinae were then washed in 1× PBS several times and mounted using fluorescent mounting media (DAKO) and allowed to dry at RT before imaging. Images from each retinal quadrant (dorsal, ventral, nasal, and temporal) were obtained using a Nikon A1S1 confocal microscope. Confocal images were taken as a single image (not z-stack) focused at the cone cell body level. Settings were kept consistent across the red channel for all images/samples. Image processing and automated cone counts were then conducted using a script programed and run by FiJi ImageJ (RRID:SCR_002285).

### Electroretinography

The full-field ERGs of 13 mice (four WT, three homozygous Kv2.1 KO, three homozygous Kv8.2 KO, and three double homozygous Kv2.1 KO/Kv8.2 KO) were measured using a custom-built apparatus. Mice were dark-adapted overnight and handled subsequently only under dim red light. Mice were anaesthetized initially with an intraperitoneal injection of ketamine (75 mg/kg) and medetomidine (1 mg/kg), and as the entire recording protocol lasted for 1.25 h, a further injection of half that dose was given after 45 min. A Peltier element-driven heating mat and a rectal thermocouple temperature probe were used to ensure body temperature remained stable (36–38°C). The pupil of the right eye was dilated by applying 1% tropicamide (MYDRIACYL; Alcon) to the surface of the cornea, which was kept moist throughout the experiment with lubricating eye drops (Viscotears; Alcon).

The mouse was positioned with its head protruding into the center of a Ganzfeld sphere (28 cm in diameter). The interior surface of the sphere was coated in multiple layers of titanium-dioxide based matte white paint (Dulux). High-brightness white-light-emitting diodes provided background adaptation illumination (Luxeon K; Philips Lumileds) and flash stimuli (Vero 29; Bridgelux). Light intensity was controlled by adjusting the current supplied to the LEDs (through a voltage-to-current converter) and through the use of neutral density filters (Lee Filters).

The spectral irradiances of the different stimulus and adapting lights used were measured at the position of the cornea using a calibrated USB4000 spectroradiometer (Ocean Optics) and converted into spectral radiances and then photopic luminances using the appropriate CIE V(λ) luminous efficiency function.

The active electrode was a silver wire loop placed on the cornea and the reference electrode was a silver wire embedded in a saline-soaked cotton wool ball in the mouth. The mouse was grounded via a hypodermic needle inserted just beneath the skin on the flank. Corneal potentials were amplified (10,000×; AC-coupled) differentially using a DAM50 amplifier (World Precision Instruments) with high and low pass filters set at 1 Hz and 1 kHz, respectively. Amplified voltages were digitized using a USB_6353_ X-Series multifunction data acquisition device (National Instruments), which was also used to control light stimulus presentation.

A dark-adapted single-flash intensity series was obtained through presentation of 1-ms flashes with the following intensities (all in mcd⋅s m^−2^): 0.1, 0.3, 1, 2, 4, 10, 100, 1000, 3000, 5000, and 10,000. The time interval between consecutive flashes and the number of times the stimulus was repeated (for subsequent averaging) varied according to stimulus intensity and was 5 s and 20 repeats for 0.1–1 mcd⋅s m^−2^, 20 s and 10 repeats for 2–100 mcd⋅s m^−2^, and 60 s and 5 repeats for 1000–10,000 mcd⋅s m^−2^.

To assess recovery of the retina, a dark-adapted paired-flash protocol was used. An initial 5-ms probe flash of 25,000 mcd⋅s m^−2^ was followed by a second test flash of equal intensity and duration a variable time later (200–6000 ms; in 200-ms increments from 200 to 2000 ms and 400-ms increments from 2000 to 6000 ms), with a 30-s interval between each probe-test pair.

To assess the temporal response of the retina, the mice were then light-adapted for at least 5 min at a background luminance of 50 cd m^−2^ before presentation with trains of 1-ms flashes of 10,000 mcd⋅s m^−2^ intensity at either 1 Hz (10 cycles, 1 s between repeats, repeated 10 times) or 20 Hz (60 cycles, 1 s between repeats, repeated 10 times).

### Experimental design and statistical analysis

The ERG and OCT analyses were based on four WT mice and three of each KO genotype. All analyses were conducted during a single session on the same group of mice. WT mice were normal littermates to the KO mice, two from the Kv8.2 line and two from the Kv2.1 line. No differences between WTs were observed. H & E images were selected from slides derived from six eyes (three WT and three Kv8.2 KO), and TUNEL quantifications were obtained from three eyes for each genotype (WT and Kv8.2 KO). Cone quantification from retinal flat mounts used images from four Kv8.2 KO and five WT animals. Means and SEs were calculated using Excel built-in functions. Paired *t* tests for comparisons were made using the on-line packages at www.graphpad.com/quickcalcs/ttest1.cfm (GraphPad, RRID:SCR_000306).

## Results

Our studies have focused mostly on female mice at six months of age; this age was selected to ensure that sufficient time had elapsed for any clinical phenotype to develop.

### Retinal morphology

**Figure 2.**
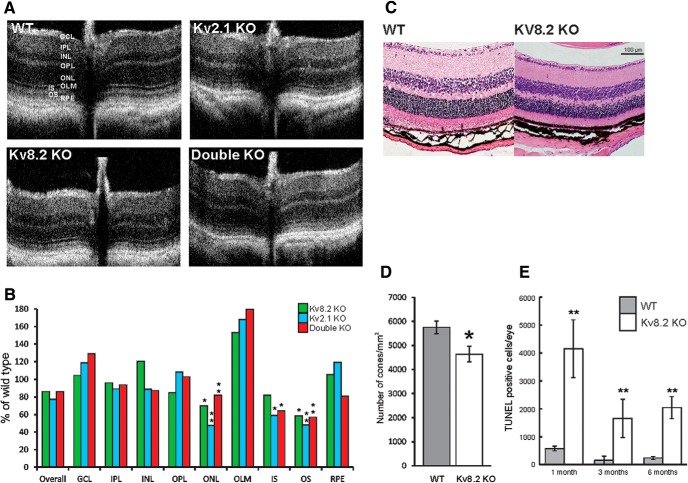
Retinal images and analysis of retinal layers and cell death. ***A***, OCT images at six months of age. ***B***, H & E images of transverse sections of WT and Kv8.2 KO retinae at six months of age. Scale bar = 100 µm. ***C***, Thickness of retinal layers at six months of age, expressed as a % of WT. Kv8.2 KO, black; Kv2.1 KO, white; double KO, hatched. GCL, ganglion cell layer; IPL, inner plexiform layer; INL, inner nuclear layer; OPL, outer plexiform layer; ONL, outer nuclear layer; OLM, outer limiting membrane; IS, inner segments; OS, outer segments; RPE, retinal pigmented epithelium. Kv8.2 KO, black; Kv2.1 KO, white; double KO, hatched. ***D***, Cone cell loss in WT and Kv8.2 KO retinae at six months of age. ***E***, TUNEL-positive cells in the retinae of WT (gray) and Kv8.2 KO (white) mice at three ages. Error bars indicate SEM; * and ** denote significance at the 5% and 1% probability level, respectively.

The optic nerve was used for orientation of OCT imaging of the retinae in live mice at six months of age. This revealed that the main layers of the retina are clearly defined in all three KO genotypes (Fig. 2*A*). Although the overall thickness of the retina was reduced, only the photoreceptor layers, that include the outer nuclear layer (ONL), showed significant thinning to around 60% of WT (Fig. 2*B*), although outer segments appear intact. This was confirmed by light microscopy of H & E-stained sections of six-month-old WT and Kv8.2 KO retinae (Fig. 2*C*).

As the human disorder presents as a cone dysfunction, the number of cones was determined at six months of age in flat mounts of Kv8.2 KO retinae by immunohistochemistry using a cone-specific arrestin antibody. As shown in Figure 2*D*, cone numbers are reduced at six months to around 80% of WT, with no significant differences in cone reduction between different retinal areas. This contrasts with the overall reduction in the photoreceptor layers of around 60%, which indicates that there is a greater loss of rods than cones.

TUNEL labeling was used to quantify cell death in the retina of WT and *Kv8.2* KO mice. As shown in Figure 2*E*, the number of TUNEL-positive cells in the Kv8.2 KO compared to WT at one, three, and six months of age, respectively, was 7-, 11-, and 9-fold higher, although the total number of TUNEL-positive cells was lower in both WT and KO mice at the older ages.

### Electroretinography, scotopic responses

**Figure 3.**
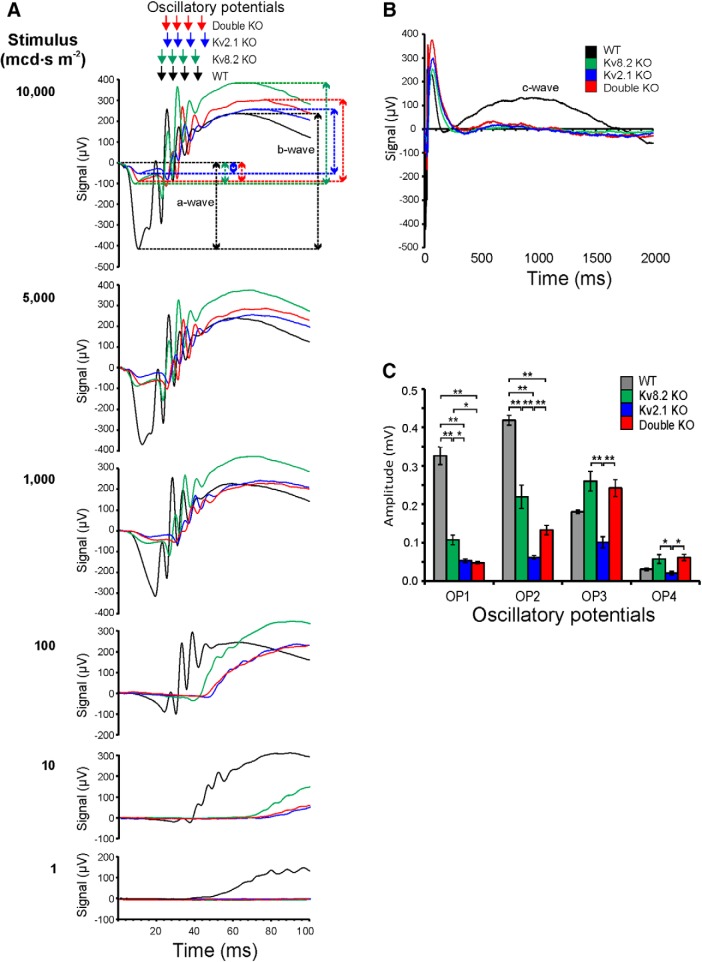
ERG traces for WT and KO mice. ***A***, a- and b-waves arising from 1-ms flashes at six stimulus intensities of 1, 10, 100, 1000, 5000, and 10,000 mcd⋅s m^−2^. Dotted lines and double-headed arrows indicate the a- and b-wave amplitudes at 10,000 mcd⋅s m^−2^. Top panel also shows comparative positions of OPs arising from 1-ms flashes at a stimulus intensity of 10,000 mcd⋅s m^−2^. The position of the four OPs are indicated by single arrows. ***B***, c-wave arising from 1-ms flashes at 10,000 mcd⋅s m^−2^. ***C***, Amplitudes of OPs. Error bars indicate SEM; * and ** denote significance at the 5% and 1% probability level, respectively.

Dark-adapted mice were subjected to a regimen of increasing flash intensities at 0.1, 1, 4, 10, 100, 1000, 3000, 5000, and 10,000 mcd⋅s m^−2^. Figure 3*A* shows the ERGs traces for six of these nine flash intensities used; the amplitudes of the a- and b-wave responses in the WT and KO mice at 10,000 mcd⋅s m^−2^ are indicated by arrows and dotted lines. An a-wave is clearly present in the ERG responses of WT mice at 100 mcd⋅s m^−2^, whereas it is only clearly discernible at 1000 mcd⋅s m^−2^ and above in the KO mice. Note also that at 5000 and 10,000 mcd⋅s m^−2^, the a-wave in WT comprises a double peak, but this is absent in KO mice. A b-wave can be clearly seen at 10 mcd⋅s m^−2^ in WT mice but is only present in KO mice from 100 mcd⋅s m^−2^ and above. Finally, a distinct c-wave is present in WT mice from 1000 mcd⋅s m^−2^ and above (Fig. 3*B*) but is completely absent in all KO mice, irrespective of stimulus intensity.

The shape of the a-wave of the KO mice differs from that of WT; in WT, the a-wave trough at 9.6 ms after the initial stimulus is followed by a small negative spike at 15 ms, but the latter is completely absent in the KOs (Fig. 3*A*, top panel). Thereafter, there are four major oscillatory potentials (OPs) generated in WT and in KO mice (Fig. 3*A*, top panel); in the KO mice, the first spike always has a larger negative amplitude than the a-wave whereas in WT, this is reversed. The timing of the OPs is very similar in WT, Kv8.2 and double KO mice (Fig. 3*A*, top panel), but slightly delayed in the Kv2.1 KO. The amplitude of the oscillations however varies considerably across genotypes (Fig. 3*C*), with OP1 and OP2 being substantially larger in WT than in KO mice, although the amplitude in the Kv8.2 KO is substantially higher than in the Kv2.1 KO and double KO. For OP3 and OP4, the amplitudes are more variable across genotypes. The loss of functional channels, as found in the Kv2.1 KO and double KO mice, would appear therefore to cause a greater reduction in the amplitude of OP1 and OP2 than in Kv8.2 KO mice where unmodulated but functional channels may be present.

**Figure 4.**
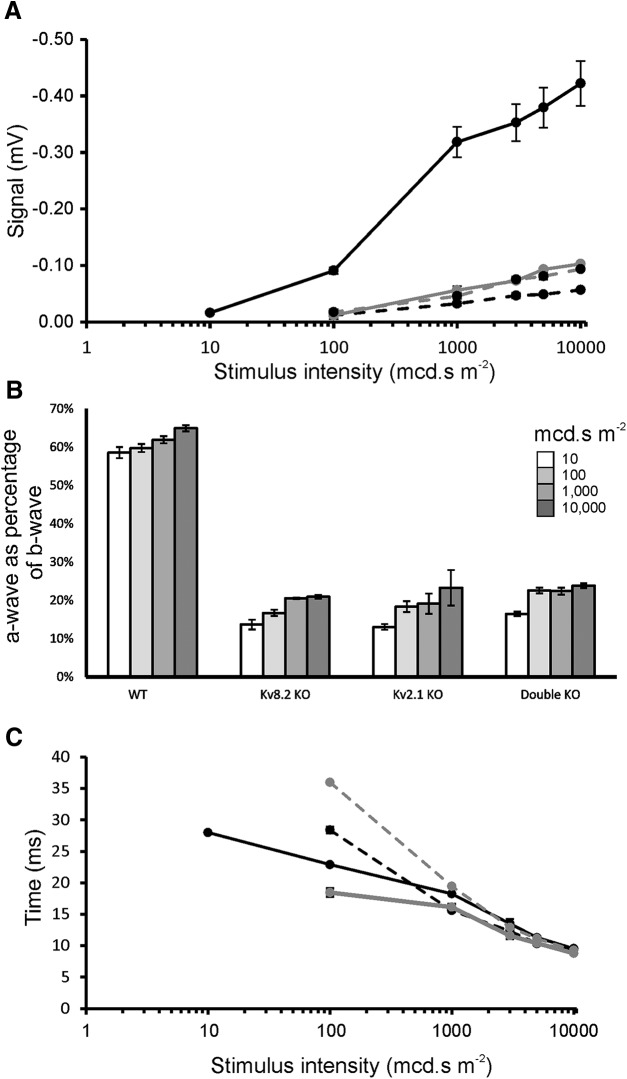
Amplitudes and implicit times for a-wave. ***A***, a-wave amplitudes. ***B***, Contribution of a-wave to b-wave amplitude. ***C***, Implicit times for a-wave. WT, solid black line; Kv8.2 KO, solid gray line; Kv2.1 KO, dashed black line; double KO, dashed gray line. Error bars represent ± SEM.

The a-wave amplitude is severely depressed in all three KO genotypes at all stimulus intensities, both in absolute terms (Fig. 4*A*) and when expressed as a percentage of b-wave amplitude (Fig. 4*B*). Since Kv channels are totally absent in double KO mice, this implies that the a-wave response in these mice does not require Kv channels and, since the a-wave amplitudes are very similar across all the KO genotypes, this also implies that Kv channels are not functional in Kv2.1 KO and Kv8.2 KO mice. In contrast, the a-wave implicit times (Fig. 4*C*) for the three KOs, although somewhat disparate at 100 mcd⋅s m^−2^, are thereafter very similar to WT and follow an identical pattern to WT of a-wave reduction in time as the flash intensity increases.

**Figure 5.**
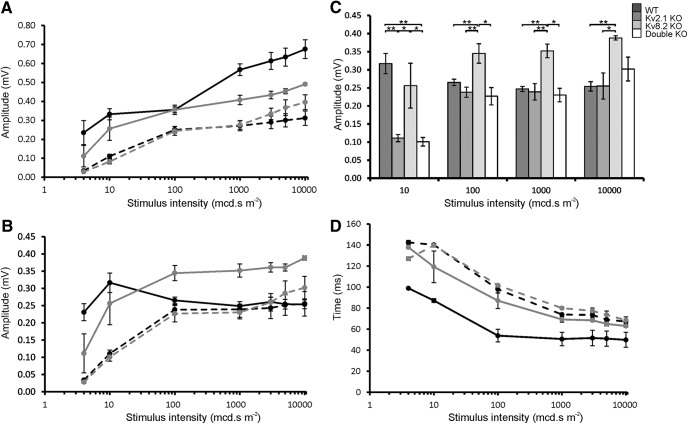
Amplitudes and implicit times for b-wave. ***A***, b-wave amplitudes. ***B***, Peak values of the positive component of b-wave. ***C***, Peak values of b-wave response at four stimulus intensities showing. ***D***, Implicit times for b-wave. WT, solid black line; Kv8.2 KO, solid gray line; Kv2.1 KO, dashed black line; double KO, dashed gray line; ** and * denotes significance at 1% and 5% probability levels, respectively. Error bars represent SEM.

A b-wave is first discernible in the ERG response of WT mice at 4 mcd⋅s m^−2^, increasing in amplitude thereafter with increasing stimulus intensity (Fig. 5*A*). In the Kv8.2 KO, a b-wave is also present at 4 mcd⋅s m^−2^, although very delayed, whereas it is barely detectable at this stimulus intensity in Kv2.1 KO and double KO mice. The b-wave amplitudes remain significantly depressed in the Kv2.1 KO and double KO mice across all stimulus intensities whereas, for the Kv8.2 KO, the amplitude is similar to WT at flash intensities up to 100 mcd⋅s m^−2^. Thereafter, as the flash intensities increase, the amplitudes in the Kv8.2 KO become less than in WT but remain significantly above those of the Kv2.1 KO and double KO. Unlike CDSRR patients therefore, the Kv8.2 KO mouse fails to show an enhanced b-wave. However, if the amplitudes of the responses above the pre-stimulus baseline are considered alone (Fig. 5*B*), it is evident that these peak values are elevated above WT in the Kv8.2 KO and are similar to WT in the Kv2.1 KO and double KO. When expressed as a percentage of the b-wave, the a-wave in WT ranges from 58% to 65% depending on stimulus intensity, whereas in the KO mice, it ranges from a low of 3% in Kv8.2 KO mice at 100 mcd⋅s m^−2^ to a maximum of only 24% in double KO mice at 10,000 mcd⋅s m^−2^. It is therefore the severely depressed a-wave in KO mice that reduces the overall amplitude of the b-wave and prevents an enhanced rod b-wave response in Kv8.2 KO mice.

The differences between b-wave peak amplitudes in WT and KO mice were examined in more detail at four stimulus intensities, 10, 100, 1000, and 10,000 mcd⋅s m^−2^. As shown in Figure 5*C*, the response is significantly enhanced in the Kv8.2 KO compared to WT and to all but one of the KOs at the three higher stimulus intensities, the exception being the double KO at 10,000 mcd⋅s m^−2^. In contrast, at the lowest intensity of 10 mcd⋅s m^−2^, the peak value in WT is significantly higher than in the Kv2.1 KO and double KO, whereas it is similar to WT in the Kv8.2 KO, and like WT, this latter response is significantly higher than for the Kv2.1 KO and double KO. From these results, it would appear that the response in the Kv2.1 KO and double KO is always below that in the Kv8.2 KO. Since Kv2.1 subunits are absent in both the Kv2.1 KO and double KO, the responses must be independent of Kv channels, whereas the response in the Kv8.2 KO must represent the effect of a lack of Kv8.2 modulation on active Kv channels. At lower stimulus levels, the response is depressed but then rises to elevated levels as intensities increase. Also, the depressed response in the Kv2.1 KO and double KO indicates that this Kv channel-independent response is less sensitive at lower light levels but rises to the WT level as light intensities increase.

For WT and KO mice, the implicit times to peak amplitude of the b-wave show a progressive reduction with increasing flash intensities (Fig. 5*D*). The responses are however always significantly delayed in KO mice compared to WT, which implies that Kv2.1 homomeric channels, as potentially present in Kv8.2 KO mice, do not compensate for the total loss of functional channels as found in the Kv2.1 KO and double KO.

### Dual flash recovery

The time interval required for the recovery of photoreceptor sensitivity is directly linked to the processes of phototransduction (Fain et al., 2001). A series of dual flash experiments were conducted to assess the recovery of a- and b-wave responses and thereby any direct involvement on rod phototransduction. An initial 5-ms probe flash of 25,000 mcd⋅s m^−2^ was followed by a second test flash of equal intensity and duration at intervals of 200 ms up to 2000 ms, and thereafter at 400-ms increments to 6000 ms.

**Figure 6.**
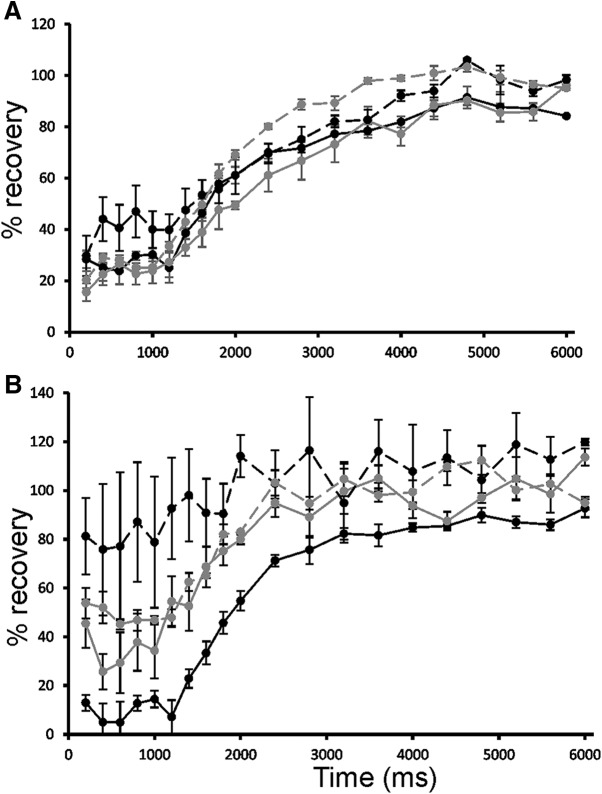
Dual flash recoveries. ***A***, b-wave recovery. ***B***, a-wave recovery. An initial 5-ms probe flash of 25,000 mcd⋅s m^−2^ was followed by a second test flash of equal intensity and duration at increasing intervals from 200 to 6000 ms, with a 30-s interval between each probe-test pair. WT, solid black line; Kv8.2 KO, dashed black line; Kv2.1 KO, solid gray line; double KO, dashed gray line. Error bars represent SEM.

For WT, the a-wave shows a recovery to 70% of initial response after 2400 ms, and a slower recovery thereafter (Fig. 6*A*). In the KO mice, recovery is more rapid, especially for the Kv2.1 KO where 80% recovery is seen after only 200 ms. Since the small a-wave seen in KO mice does not require Kv channels, this implies that the recovery of this non-Kv channel component is very rapid and most likely not linked to the recovery of phototransduction. In contrast, the b-wave recoveries showed a similar time response in WT and KO mice (Fig. 6*B*).

### Electroretinography, photopic response

**Figure 7.**
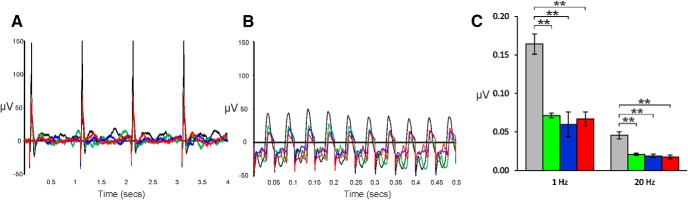
Photopic response in mice light-adapted at a background luminance of 50 cd m^−2^ before exposure to trains of 1-ms flashes of 10,000 mcd⋅s m^−2^ intensity at (***A***) 1 Hz (10 cycles, 1 s between repeats, repeated 10 times) and (***B***) 20 Hz (60 cycles, 1 s between repeats, repeated 10 times). ***C***, Amplitudes at 1- and 10-Hz frequencies. WT, gray; Kv8.2 KO, green; Kv2.1 KO, blue; double KO, red; ** denotes significance at 1% probability level. Error bars represent SEM.

To assess cone sensitivity, mice were light-adapted at 50 cd m^−2^ with a separate calibrated LED light source that was on for at least 5 min before stimuli were presented and remained on for the duration of the test. Trains of 1-ms flashes of 10,000 mcd⋅s m^−2^ intensity were presented at either 1 Hz (10 cycles, 1 s between repeats, repeated 10 times) or 20 Hz (60 cycles, 1 s between repeats, repeated 10 times). As shown in Figure 7, the KO genotypes all show significant reductions in amplitude at both stimulus frequencies compared to WT. There was, however, no major delay in response, although all KO mice were delayed by 2–4 ms compared to WT in the time to first peak of the flicker wave form.

## Discussion

The ERG wave form is composed of three major components: PI, PII and the PIII fast/slow complex (Witkovsky et al., 1975; Granit, 1933). The corneal-negative a-wave reflects the leading edge of the negative fast PIII component. Its amplitude is measured from the pre-stimulus baseline to the most negative trough of the ERG (McCulloch et al., 2015). The positive b-wave corresponds to the PII component; b-wave amplitude is calculated as positive deflection from the trough of the a-wave. The a-wave of the ERG reflects the activity generated by the photoreceptors (Hood and Birch, 1993; Sieving et al., 1994) whereas the b-wave is derived from the inner retina, predominantly from Müller and ON-bipolar cells (Miller and Dowling, 1970). The positive c-wave is the summation of the positive PI and the negative slow PIII components. It is derived from the RPE and reflects the change in the transepithelial potential due to the hyperpolarization at the apical membrane of the RPE cells and the hyperpolarization of the distal portion of the Müller cells. Finally, OPs represent neural activity that is distinct from that of the a- and b-waves.

A role for voltage-gated K^+^ channels in the visual process has been known for some time (Barnes, 1994) but the direct demonstration of a role for channels incorporating the Kv8.2 subunit in this process arose from the identification of mutations in the *KCNV2* gene in human CDSRR (Wu et al., 2006). The unique ERG phenotype seen in patients with CDSRR appears to be linked to the loss of the modulatory role of Kv8.2 subunits in voltage-gated heterotetrameric channels with Kv2.1 subunits (Czirják et al., 2007; Smith et al., 2012).

Two mouse KO models have been used in this study: a deletion and insertion mutation of *Kcnb1* that renders the resulting transcript non-functional in terms of coding for the Kv2.1 subunit (Jacobson et al., 2007), and a deletion mutation of the *Kcnv2* gene that results in the absence of Kv8.2 subunits and generates therefore a true null mutation. Kv2.1 KO mice suffer from epileptic-like seizures (Speca et al., 2014) but this is not seen in the Kv8.2 KO, although missense mutations in *KCNV2* have been shown to cause epilepsy in humans (Jorge et al., 2011).

Similar to the changes reported in CDSRR patients (Zobor et al., 2012), histologic analysis of the retinae of Kv8.2 KO mice revealed a mild reduction in overall thickness which is largely attributable to a reduction in the ONL. Cell death in the retina, as determined by TUNEL analysis, was substantially higher than WT in the Kv8.2 KO retina, with increases ranging from 7- to 11-fold depending on age. Overall cone loss at only 20% is however relatively mild, indicating a greater loss of rod photoreceptors to account for the thinning of the retina and the higher frequency of cell death. Similar changes have been reported in CDSRR patients, although in more severe cases, the inner/outer segment border is less defined (Zobor et al., 2012). Such limited changes in retinal structure and integrity would not be expected by themselves to result in such severe reductions in light sensitivity, as revealed by the depressed ERGs at low flash intensities seen in all three KO genotypes. These latter changes imply therefore that the loss of the K^+^ channel subunits has a direct physiologic impact on the visual process.

The scotopic a-wave is considered to be a direct reflection of the rod photocurrent (Breton et al., 1994) and, as modeled by Lamb and Pugh (1992), is thought to arise from the activation of the phototransduction cascade. The severely reduced a-wave seen in all the KO mice models demonstrates that voltage-gated K^+^ channels are a major contributor to the a-wave and that heteromeric Kv channels are required for a normal amplitude. Significantly, the reduction in a-wave amplitude is similar across all KO mice. Kv2.1 KO and double KO mice lack Kv2.1 subunits and hence the capacity to produce Kv channels, whereas the Kv8.2 KO, which retains an intact *Kcnb1* gene, possesses the capacity to synthesize Kv2.1 subunits. This result implies that homomeric Kv2.1 channels in the Kv8.2 KO do not contribute to the a-wave response. It also identifies two components to the a-wave, a small component that is retained in KO mice that does not require functional Kv channels, and a second larger component seen in WT mice that is Kv channel dependent; a normal a-wave is therefore the summation of these two components. It is interesting that the a-wave shows a much more rapid recovery in dual flash experiments in KO mice than in WT, indicating that the small, non-Kv channel-dependent a-wave in these mice has a more rapid rate of recovery than the Kv channel-dependent one.

The reduced amplitude of the a-wave in KO mice would be expected to be correlated with a reduced activation of the phototransduction cascade but the presence of relatively normal b-wave amplitudes seen at higher flash intensities would suggest that this is not the case. This apparent disconnect between the amplitudes of the a- and b-waves in KO mice may be attributable to the loss of the larger Kv channel-dependent component of the a-wave. Note that the normal rate of recovery of the b-wave in KO mice indicates that the phototransduction cascade itself is not primarily affected. A similar conclusion has been reached for patients with CDSRR (Wissinger et al., 2008). In CDSRR, the amplitude of the a-wave is also reduced or even absent at lower flash intensities but unlike KO mice, may approach normal amplitudes at higher intensities (Gouras et al., 1983; Tanimoto et al., 2005; Wissinger et al., 2008; Sergouniotis et al., 2012; Zobor et al., 2012; Fujinami et al., 2013; Grigg et al., 2013; Vincent et al., 2013). The implicit times for the generation of the much-reduced a-wave in KO mice are similar to WT and, like WT, show a progressive reduction in time as flash intensities increase. In contrast, in CDSRR, the a-wave implicit time is generally delayed (Zobor et al., 2012).

The presence of a b-wave in Kv2.1 KO and double KO mice implies that Kv channels are not required for a b-wave to be generated. As found for the a-wave, the amplitude of the b-wave is severely depressed at lower flash intensities in all KO mice compared to WT, but at higher flash intensities, the amplitudes are substantially higher; in Kv2.1 KO and double KO mice, the increase in b-wave amplitude is progressive from 10 mcd⋅s m^−2^ but always remains below WT, even at the highest stimulus used, whereas in Kv8.2 KO mice, the increase from 10 mcd⋅s m^−2^ is much more rapid, reaching an amplitude that almost matches the WT response at 100 mcd⋅s m^−2^. It does not however exceed the WT amplitude. It should be noted however that a “supernormal rod response” at higher flash intensities is not always evident in CDSRR patients (Zobor et al., 2012) and it has been suggested that the disorder should be referred to as “*KCNV2* retinopathy” (Sergouniotis et al., 2012). It is also important to note that it is the severe reduction in the a-wave in KO mice that limits the amplitude of the b-wave; at the highest flash intensity used, the a-wave component of the ERG accounts for around 66% of the b-wave in WT mice but only around 20% in the KO mice. This translates into a positive component above the pre-stimulus baseline of the b-wave that is similar to WT in the Kv2.1 KO and double KO mice but substantially higher than WT in the Kv8.2 KO. This latter component is therefore not dependent on the presence of Kv channels but where functional channels are present as in WT, the response is capable of modulation. When this modulation is absent, as in the Kv8.2 KO, the b-wave response becomes enhanced at higher stimulus intensities. In contrast, the similarity in the peak amplitudes above pre-stimulus baseline of the Kv2.1 KO and double KO to WT indicates that this level of response does not require active Kv channels, as they are missing in both KOs; the b-wave in these KO mice, which is severely depressed at lower stimulus levels but rises at higher intensities, must be independent of the presence of functional Kv channels.

The generation of the b-wave in KO mice shows a similar delay in all three KO genotypes compared to WT, with the implicit time reducing in parallel with WT as the stimulus intensity increases but always remaining delayed. In this regard, the Kv8.2 KO is similar to CDSRR patients where the implicit time for the b-wave is significantly delayed.

The c-wave component of the ERG is known to originate in the pigment epithelium and to be linked to the light-induced reduction in extracellular K^+^ concentration that results from photoreceptor activation (Witkovsky et al., 1975; Oakley and Green, 1976). The absence of a c-wave in all three KO genotypes indicates that the movement of K^+^ out of the photoreceptors via these channels is more-or-less eliminated, even in Kv8.2 KO mice with the potential to produce homomeric Kv2.1 channels. It would appear therefore that homomeric Kv2.1 channels alone do not result in a permanent outward K^+^ current.

It has been suggested in a recent study (Gayet-Primo et al., 2018) that Kv2.1 homomeric and Kv2.1/Kv8.2 heteromeric channels exist as two distinct populations of Kv channels in photoreceptors, with homomeric channels responsible for a high voltage-activated current and heteromeric channels for a low voltage-activated current. This latter current has been equated to the *I_KX_* current directly involved in the setting of the dark resting potential and acceleration of the voltage response to small photocurrents (Beech and Barnes, 1989). Heteromeric channels are absent from Kv8.2 KO mice, so the *I_KX_* must also be absent and, as discussed above, homomeric Kv2.1 channels appear to be non-functional in the generation of an a-wave, so the high voltage-activated current is lost in Kv2.1 KO mice. When both Kv2.1 and Kv8.2 subunits are absent, as in the double KO mouse, both the high and low voltage-activated currents would be lost.

For the photopic response, all KO genotypes show a consistent reduction in flicker amplitude compared to WT. What is distinctly different is that the significant flicker delay seen in CDSRR patients (Vincent et al., 2013) is not present in the mouse KOs where there is a 2- to 4-ms delay at most. The reason for this is not entirely clear but may be related to the absence of Müller cells in the human fovea (and the buffering effect on extracellular K^+^ that they provide), which makes cones more vulnerable (Zobor et al., 2012) or inherent differences in the architecture of the mouse and human retina. This is supported by the observation that the Kv8.2 KO mouse retains around 80% of cones by six months of age, which if this is also the case for CDSRR patients, suggests that this disorder may be amenable to relatively late treatment. The delayed responses present in CDSRR patients may reflect therefore an impact on cones that is associated with the presence of a cone-rich fovea and lack of Müller cells in this region of the human retina.

Patch-clamp studies have identified up to 5 different ionic currents in the inner segments of rod photoreceptors which includes a mixture of Ca^2+^-dependent (*I*_Cl(Ca)_ and *I*_K(Ca)_), and voltage-dependent currents (*I*_Kx_, *I*_h_, and *I*_Ca_; MacLeish and Nurse 2007). Dissecting the separate currents, channels and their individual contribution in the overall electrophysiological response of rod photoreceptors is still very much a work in progress and will require a multidisciplinary approach outside the scope of the present study. To elucidate the exact mechanism and contribution of the Kv2.1/Kv8.2 voltage-gated K^+^ channels to the formation of the a- and b-waves, future work will be necessary and could extend to the testing of voltage-gated K^+^ channel blockers, patch-clamping recordings of photoreceptors from the Kv KO retinas and side-by-side comparison of the electrophysiological response of KO models of the different ionic channels.

In summary, the study of the ERG profiles in Kv2.1 KO and Kv8.2 KO mice presented here has progressed our understanding of the role of Kv channels in the generation of a- and b-waves. The scotopic ERG displayed by the Kv8.2 KO mouse shows many similarities to CDSRR in humans. In particular, the amplitude of the a-wave component of the ERG is severely depressed, as is the amplitude of the b-wave at dim light levels, but this then shows a rapid rise to approximate WT levels at higher flash intensities. Nevertheless, it is only the reduced contribution of the a-wave that prevents the production of a supernormal rod ERG. Furthermore, the Kv2.1 KO also has a retinal phenotype of a depressed ERG, but lacks any enhanced response. However, no human retinal disorders arising from mutations in *KCNB1* have yet been reported. Comparisons between the a- and b-waves of WT and KO mice shows that both comprise Kv dependent and independent components.

## Synthesis

Reviewing Editor: Siegrid Löwel, University of Goettingen Decisions are customarily a result of the Reviewing Editor and the peer reviewers coming together and discussing their recommendations until a consensus is reached. When revisions are invited, a fact-based synthesis statement explaining their decision and outlining what is needed to prepare a revision will be listed below. Note: If this manuscript was transferred from JNeurosci and a decision was made to accept the manuscript without peer review, a brief statement to this effect will instead be what is listed below.

This is a manuscript transferred from JNS. The authors have addressed a number of minor typographical and formatting edits suggested by the JNS-reviewers. In addition, they have also expanded the discussion to respond to other important comments of the previous reviewers. However, figure 3 does not seem to be improved according to Reviewer 2's suggestions: The superimposition of multiple ERG-traces makes it really difficult to differentiate traces from one another. Therefore I ask the authors to re-work this figure, and also enlarge the numbers on the y-axes.

## Author Response

Livia Carvalho, PhD

Lions Eye Institute, University of Western Australia

2 Verdun Street

Nedlands, WA 6009, Australia

+61 (8) 93810712

liviacarvalho@lei.org.au

Prof. Siegrid Löwel

Reviewing Editor

eNeuro

February 01st 2019

Dear Prof. Siegrid Löwel

I am pleased to re-submit the edited version of our research manuscript “The role of the voltage-gated potassium channel proteins Kv8.2 and Kv2.1 in vision and retinal disease: insights from the study of mouse gene knock-out mutations” by Nathan Hart and colleagues for publication in eNeuro.

We have now edited the manuscript and figure 3 according to the suggestion of the reviewers and requested by the reviewing editor. In the new version of figure 3 we have separated the ERG traces from panel A into individual panels per stimulus intensity. We have also deleted panel C showing the oscillatory potentials from the previous version as the same information is now shown in the top panel graph in A. Panel D is now C. Numbers on the Y axes have been enlarged to provide clearer information.

In the new submitted manuscript versions (edited showing in red our changes and a clean copy with title page) we have modified the legend for figure 3 and the text in page 10 where this figure is referenced.

We hope the changes are to the satisfaction of the reviewers and provides an improvement to our manuscript.

Thank you for your consideration.

Sincerely,

Livia Carvalho, PhD

Group Leader/Senior Research Fellow

Retinal Genomics and Therapy, Lions Eye Institute

University of Western Australia
